# Multi-Generational Perspectives on the Competencies Required of First-Line Nurse Managers: A Phenomenological Study

**DOI:** 10.3390/ijerph191710560

**Published:** 2022-08-24

**Authors:** Pin-Pin Choi, Suet-Shan Wong, Wai-Man Lee, Mei-Ha Tiu

**Affiliations:** 1 School of Nursing and Health Studies, Hong Kong Metropolitan University, Hong Kong; 2 School of Nursing, St Teresa’s Hospital, Hong Kong

**Keywords:** management, phenomenology, professional roles, workforce

## Abstract

First-line nurse managers play an integral role in ensuring team and organizational effectiveness and quality of care. They are facing increasing challenges arising from the need to lead a generation-diverse workforce. Further research that examines multi-generational perspectives on the competencies of first-line nurse managers is warranted. This paper aimed to elucidate multi-generational perspectives on the competencies required of first-line nurse managers based on their lived experiences and perceptions, as well as those of frontline nurses. A descriptive phenomenological approach was adopted. A total of 48 informants were invited to individual semi-structured interviews to share their perspectives on the competencies required of first-line nurse managers. Findings were analyzed using Van Kaam’s controlled explication method. Four themes that described four areas of competency were generated: (1) advocating for the interests of the staff, (2) allocating resources effectively, (3) building cohesive teams, and (4) embracing change and quality. The findings indicated that there were significant discrepancies among the different generations of informants in terms of their degree of commitment to work, preferred modes of team communication and collaboration, and perspectives on the role and function and preferred leadership styles of first-line nurse managers. This study fell short in examining the experiences of Generation Z nurses, and the findings are subject to further validation by larger samples. However, this study has implications for hospital administrators, nurse educators, and managers, encouraging them to rethink the notion of management competencies to develop effective strategies for leading a multi-generational workforce.

## 1. Introduction

Over the past decade, attention has been increasingly paid to the role of first-line nurse managers in ensuring organizational effectiveness and quality of care. The work of first-line nurse managers has been regarded as the most challenging among all levels of nurse leaders because of the proximity of first-line nurse managers to the point of care. They must take on both administrative and clinical duties, manage unexpected situations in the frontline setting, and struggle with the competing demands of frontline staff and the organization [1,2]. However, studies on first-line nurse managers are scant when compared to those on nurse executives and nurse administrators.

Previous studies on the competencies of first-line nurse managers, namely the attributes and behaviors essential for managerial competence, varied in their intensity and scope. Most were confined to a single type of competency, for instance the caring [3], informatics [4], evidence-based practice competencies [5], the competencies critical to effective communication [6], and staff retention [7]. Other studies were conducted by sampling all levels of nurse leaders, including those in the top, middle, and first levels [8,9]. A few scholars pointed out that the competencies crucial for managerial effectiveness can vary significantly across different levels of management; therefore, there is no one set of generic competencies that would apply to all levels of nurse leaders [10,11].

Contemporary researchers have reviewed past relevant studies that examined the competencies required of first-line nurse managers and identified a wide range of factors influencing their managerial practices. These include organizational factors such as human resource and information management systems, role factors such as role preparation and demand, skill factors such as communication and collaboration skills, and personal characteristics such as personal and professional accountability [10,12,13]. An exhaustive list of competency dimensions and standards can be found in the extant literature, for instance, the “nurse manager competencies” developed by the American Organization of Nurse Executives (AONE) and the competency inventories developed by different researchers [8,14,15]. Critics have pointed out that the major factors determining the managerial success of first-line nurse managers remain unclear and inconclusive [10,12,16].

Some scholars further highlighted the need to examine the influence of generational diversity on the managerial effectiveness of first-line nurse managers [16,17]. There are four generations in the present nursing workforce, namely Baby Boomers (1946–1964), Generation X (1965–1980), Millennials (1981–2000), and Generation Z (born after 2000) [18]. Different generations of nurses are known to have their unique characteristics, values, and beliefs, which were shaped by the social, economic, political, and cultural issues that took place during the critical stages of their development. Therefore, first-line nurse managers of different generations are likely to demonstrate different management styles, while frontline nurses of different generations also tend to share different views on what contributes to managerial success [19,20]. As first-line nurse managers play a crucial role in ensuring team and organizational effectiveness, it is essential to take into account generational diversity when examining the required competencies of first-line nurse managers [19].

## 2. Aim

This paper aimed to elucidate multi-generational perspectives on the competencies required of first-line nurse managers based on their lived experiences and perceptions and those of frontline nurses.

## 3. Methods

### 3.1. Design

A descriptive phenomenological approach was adopted. Phenomenologists believe that the essence of a phenomenon or an issue can only be captured through acquiring a rich description of the lived experiences and perceptions of individuals [21]. This study involved a process of identifying crucial elements of the competencies of first-line nurse managers by acquiring a detailed description of their lived experiences and perceptions and those of their subordinates, namely the frontline nurses.

### 3.2. Setting and Subjects

This study was conducted from October 2020 to November 2021. A total of 48 informants were recruited through the research team’s personal networks; these comprised 29 first-line nurse managers and 19 frontline nurses working in public and private general hospitals in Hong Kong. The maximum variation sampling technique was adopted to sample informants with widely varying personal and work-related characteristics to expand the variety of perspectives accounted for [21]. These included first-line nurse managers and frontline nurses of different age groups and from different generations, and those with varying years of work experience and who were working in different hospitals and work settings. Frontline nurses at different ranks, comprising Registered Nurses, Enrolled Nurses, and Advanced Practice Nurses, were sampled. Informants with less than one year of work experience were excluded from the study. The first-line nurse managers were recruited from 15 hospitals and 25 work units, and the frontline nurses were recruited from 11 hospitals and 16 work units. Table 1 presents the characteristics of the informants.

### 3.3. Data Collection

Individual semi-structured interviews were conducted with the 48 informants. The interviews lasted from 50 to 110 min and were conducted face-to-face or via videoconference in a private meeting room or a place convenient to the informants. For each interview, the informant was first invited to share their lived experience of working as or with the first-line nurse manager. Subsequent questions were open-ended ones developed based on the informants’ initial responses and were related to specific aspects of their work relevant to the required competencies of first-line nurse managers. Table 2 presents the interview guide, which was pilot tested with the first two informants of this study. All of the interviews were audio-recorded and transcribed verbatim for analysis. Data saturation was achieved. It was ascertained that further interviews would not result in any new information or generate any new themes and that a complete understanding of the study issue had been obtained [21]. Ethical approval was obtained from a university (ethical approval code: HE-RGC2019/31). Informants were fully informed about the aim of and process involved in the study and their right to confidentiality and to withdraw from the study. Written consent was obtained to indicate that their participation in the study was voluntary. Gift vouchers were provided to informants as compensation for their time.

### 3.4. Data Analysis

**Figure 1:**
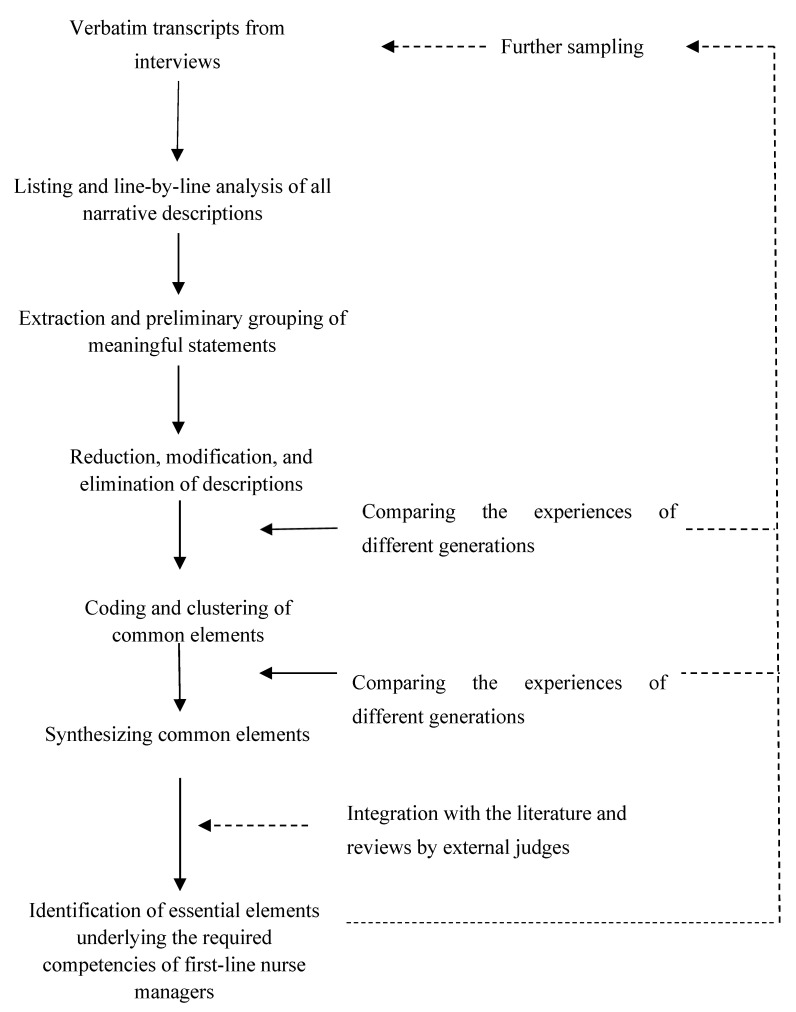
Processes involved in the phenomenological analysis.

Interview transcripts were analyzed using Van Kaam’s controlled explication method. It involves the processes of (1) immersing oneself in the phenomenological texts; (2) listing and categorizing meaningful descriptions; (3) reducing and eliminating overlapping and redundant descriptions; (4) clustering and deriving common elements from the descriptions; and (5) confirming the common elements by asking external judges to validate the phenomenological analysis [22]. Comparisons of the lived experiences and perceptions of the informants were constantly made, particularly between those of the first-line nurse managers and frontline nurses of different generations, so as to identify commonalities and differences in their viewpoints concerning what constitutes the competencies required of first-line nurse managers. Themes that described the competency areas of first-line nurse managers were generated through the constant comparative process. Interview transcripts were read and reread to ensure that data were saturated and that the findings could fully represent the views of the informants. The trustworthiness of the study was ensured by practicing reflexivity and by, involving multiple researchers and external judges and having them analyze the data independently. Throughout the course of the inquiry, the members of the research team set aside any preconceptions, prejudices, and biases that might affect the processes of data collection and analysis [21]. In addition, four external judges, including two first-line nurse managers and two frontline nurses, were engaged in validating the outcomes of the phenomenological analysis. Figure 1 summarizes the steps involved in the phenomenological analysis.

## 4. Results

**Figure 2:**
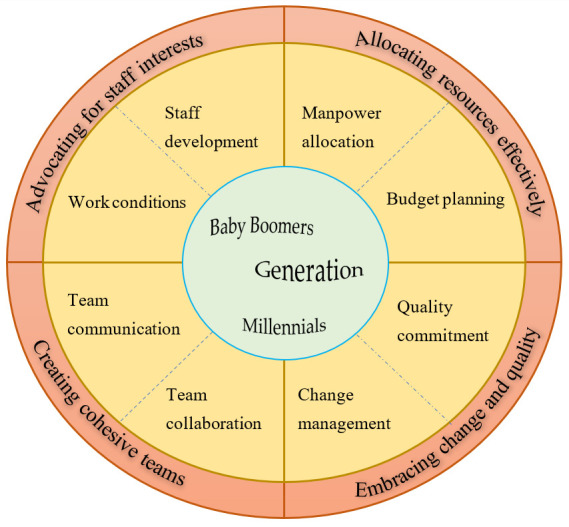
Competencies required of first-line nurse managers.

Four main themes that describe the core competency areas of first-line nurse managers were generated (Figure 2). It was noted that the first-line nurse managers and frontline nurses were in agreement on the domains in which it was crucial for first-line nurse managers to be competent. However, discrepancies were noted among informants of different generations regarding the essential elements of managerial effectiveness.

### 4.1. Advocating for the Interests of the Staff

When asked to describe their role as first-line nurse managers, most informants said that they were advocates of safeguarding the interests of frontline nurses. This was particularly the case among Baby Boomers:

“*The role of a nurse manager is being an advocate, managing the ward operation well, providing a favorable work environment for staff, speaking up for them, and defending their best interests.*” (Code M18) [Baby Boomer; Nurse Manager]

“*Being a nurse manager is about safeguarding the interests of frontline staff, relaying their appeals, and addressing their needs, so that quality of care isn’t compromised.*” (Code M22) [Baby Boomer; Nurse Manager]

While Baby Boomer nurse managers attributed their career aspirations to their role as advocates, frontline nurses who were Generation Xers and Millennials placed more weight on their personal interests and family life and were unwilling to seek promotion:

“*The workload of nurse managers is overwhelming, yet the salary is only a bit higher than that of Advanced Practice Nurses. They aren’t compensated properly for their hard work.*” (Code N02) [Generation Xer; Advanced Practice Nurse]

“*If there are emergency situations, even after working hours colleagues will call the nurse manager for follow-up. I have a family to take care of and I don’t want to spend my private time on work.*” (Code N05) [Millennial; Registered Nurse]

#### 4.1.1. Creating Favorable Work Conditions

Both frontline nurses and first-line nurse managers regarded a sense of responsibility as an important attribute for staff advocacy. Nurse managers are accountable not only to their subordinates but also to the nursing profession. These views were widely held by Baby Boomer nurse managers:

“*I feel that the younger generation of nurse managers aren’t highly committed to their work. They are unaware of their vital role in shaping the work environment, which in turn influences the work climate and quality of care.*” (Code M25) [Baby Boomer; Nurse Manager]

“*You are the person who can best represent the work unit… Whether the working conditions are good, whether staff are happy with the work environment and are willing to stay—the nurse manager is accountable for all of these matters.*” (Code M19) [Baby Boomer; Nurse Manager]

Some frontline nurses cited instances of their nurse managers defending their interests when they were treated unfairly at work:

“*Previously our colleagues were exposed to unfair treatment…. Our nurse manager did her very best to defend our interests when we faced unfair treatment. She launched a complaint to her immediate supervisor, and even to the chief executive of the hospital.*” (Code N19) [Generation Xer; Advanced Practice Nurse]

“*The hospital previously suspended the provision of a Covid special allowance for staff without notifying us. Our nurse manager tried to negotiate with the senior management about that.*” (Code N16) [Millennial; Enrolled Nurse]

#### 4.1.2. Developing Staff Potential

First-line nurse managers recognized their role in developing staff potential through offering training and development opportunities, while frontline nurses also valued forward-thinking nurse managers who gave them advice on their professional development:

“*I will spend time on making professional development plans for staff, and I see nurturing talent as my responsibility.*” (Code M24) [Baby Boomer; Nurse Manager]

“*Our nurse manager is forward-looking. He shares with us his thoughts about the future development of the nursing profession, and advises us on how we can better equip ourselves for that.*” (Code N11) [Generation Xer; Advanced Practice Nurse]

Frontline nurses further highlighted the need to be fair when coordinating training and development activities:

“*Sometimes the nurse manager conceals some information and arranges certain colleagues to attend training courses…. It’s just not fair.*” (Code N15) [Baby Boomer; Advanced Practice Nurse]

“*Our nurse manager only arranges training courses to those who have the potential to be promoted. This is totally unacceptable.*” (Code N11) [Generation Xer; Advanced Practice Nurse]

Some nurse informants further elaborated on advancing themselves through role modeling the behaviors of the nurse managers and through job delegation:

“*Our nurse manager is a role model.... I learn a lot from her, her behaviors, attitudes, and the ways in which she handles complaints from patients and their relatives.*” (Code N13) [Millennial; Advanced Practice Nurse]

“*Some colleagues are good at managing clinical-related issues. Some are good at managing procurements, and some are good at teaching. She (the nurse manager) will delegate work to us according to our strengths to develop our potential.*” (Code N17) [Generation Xer; Advanced Practice Nurse]

### 4.2. Allocating Resources Effectively

All generations of first-line nurse managers viewed resource management as a challenging part of their work. They attributed this to the nature of patient care, which involves multiple stakeholders and requires careful planning if the quality of care is to be ensured:

“*I’m still learning, particularly about managing human resources…. Our work demands quality, and involves too many stakeholders. Their characteristics must be considered when managing their work.*” (Code M23) [Generation Xer; Nurse Manager]

“*Allocating resources is the most difficult part of my work…. Every year we have to plan ahead very carefully, taking into consideration the demands on manpower and equipment, and assessing staff development needs, to ensure quality of care.*” (Code M01) [Baby Boomer; Nurse Manager]

#### 4.2.1. Ensuring Optimal Manpower Allocation

Regarding staffing issues, both frontline nurses and first-line nurse managers highlighted the role of nurse managers in ensuring adequate manpower along with the right mix of skills, as these would have implications for the quality of the care that is delivered:

“*We are short of staff now; the nurse manager has to carefully consider the mix of skills when managing staff assignments.*” (Code N03) [Generation Xer; Registered Nurse]

“*We have to be highly prudent when allocating manpower…. It would be a problem if only junior staff are on duty when critically-ill patients are admitted to the ward.*” (Code M26) [Baby Boomer; Nurse Manager]

Among the different generations of nurse managers, Generation Xers and Millennials tended to be more easily distressed by the short staffing situation and found manpower allocation and job assignments difficult to deal with:

“*Different generations of nurses collaborate with each other differently, creating different chemical actions…. It’s hard to please everyone when arranging staff assignments.*” (Code M28) [Millennial; Nurse Manager]

“*Dealing with personnel issues is difficult…. Planning the roster and allocating work duties is hard work and one can easily piss colleagues off, so I usually avoid those sorts of tasks.*” (Code M10) [Generation Xer; Nurse Manager]

#### 4.2.2. Making Effective Budget Plans

Different generations of nurse managers were cognizant of their role in making effective budget plans. When compared with Baby Boomers, Millennials were less confident in handling fiscal-related matters and were less able to describe their scope of practice and decisions made in financial planning:

“*Every year we have to plan the budget for purchasing consumable items and new instruments, and upgrading systems and facilities…. I can barely manage this.*” (Code M04) [Millennial; Nurse Manager]

“*You have to forecast what types and how many extra items and pieces of equipment will be needed in the winter surge, and how long the existing resources can last. These are trivial matters, and easy to handle.*” (Code M09) [Baby Boomer; Nurse Manager]

Frontline nurses were also aware of the crucial role played by first-line nurse managers in ensuring an adequate supply of materials, instruments, and equipment essential for the provision of care. They were, in general, satisfied with the financial competence of the nurse managers:

“*Our nurse manager knows well what the other hospitals are using, and the quality of the goods. For example, what they’re using to secure the nasogastric tubes. She can always source some quality goods for us.*” (Code N10) [Generation Xer; Advanced Practice Nurse]

“*Our nurse manager has been able to keep everything in order, even during times of renovation…. We have never encountered difficulties in locating the resources we need, as she would have sourced them beforehand.*” (Code N17) [Generation Xer; Registered Nurse]

### 4.3. Building Cohesive Teams

First-line nurse managers recognized the importance of people skills in managing team communication and collaboration. Some informants further elaborated on the challenge of creating teams because of generational diversity:

“*People skills are the most important because in our daily work we have to communicate and collaborate with different colleagues with different backgrounds and characteristics.*” (Code M24) [Baby Boomer; Nurse Manager]

“*Creating teams is never an easy task, as colleagues are from different generations. Their opinions are usually different, and conflicts easily arise when they don’t share common views at work.*” (Code M04) [Millennial; Nurse Manager]

#### 4.3.1. Ensuring Effective Communication

Regarding team communication, it was obvious that frontline nurses and first-line nurse managers have different areas of focus. Frontline nurses were more concerned about whether their views and voices were respected, while first-line nurse managers placed a greater emphasis on whether important messages about changes in policies and practices were being successfully relayed to frontline nurses:

“*I would say there is no respect between us…. I once raised a suggestion in a meeting, and my nurse manager objected without giving an explanation. He even asked me to talk to him in advance before sharing any views in future meetings.*” (Code N02) [Generation Xer; Advanced Practice Nurse]

“*There are constant changes in hospital policies; we have to relay the information to colleagues promptly…. We often reiterate important messages a few times in meetings, but colleagues are not very attentive.*” (Code M21) [Baby Boomer; Nurse Manager]

Other than the concern over a lack of open communication between the first-line nurse managers and frontline nurses, it was noted that different generations of nurses have different preferred modes of communication. Generation Xers and Millennials preferred emails and phone messages over face-to-face discussions in meetings:

“*There is no time to read documents such as protocols and guidelines in the ward. Other means of sharing information such as emails may be more effective.*” (Code N20) [Millennial; Advanced Practice Nurse]

“*Technology makes communication easier and more convenient. Personally, I think sending mass messages through mobile apps is highly efficient.*” (Code N14) [Millennial; Advanced Practice Nurse]

“*I like disseminating information and communicating with colleagues through WhatsApp. I also make use of the WhatsApp group to convey support to colleagues.*” (Code M13) [Generation Xer; Nurse Manager]

#### 4.3.2. Fostering Team Collaboration

Team collaboration in healthcare is multi-faceted and involves collaboration among different parties, including nurses, multidisciplinary members, and hospital administrators. First-line nurse managers placed high importance on their role in facilitating team collaboration to ensure the quality of care:

“*Each health profession has its unique role to play in the team. Though I’m a nurse, I’m very neutral, and I facilitate collaboration among the multidisciplinary team members to ensure that patients’ wishes can be fulfilled.*” (Code M18) [Baby Boomer; Nurse Manager]

“*In many cases, the hospital administrators and frontline nurses don’t share the same views about how things should be done…. We always play a role in mediating the conflicts and facilitating the collaboration between them and the senior management.*” (Code M02) [Baby Boomer; Nurse Manager]

Regarding the collaboration with first-line nurse managers, frontline nurses who were Generation Xers and Millennials tended to be more resentful of their nurse managers’ autocratic style of management:

“*Our nurse manager is obsessed with supreme authority. You can never challenge him, or turn him down. That’s why we deliberately keep a distance from him.*” (Code N09) [Baby Boomer; Registered Nurse]

“*She (nurse manager) commands us to strictly follow her instructions…. I believe that collaboration should be free of superiority or inferiority if we are to build a good team.*” (Code N18) [Millennial; Advanced Practice Nurse]

The need to foster team collaboration was constantly raised by Generation Xers and Millennials. Two nurse managers further shared their ways of building collaborative teams by respecting others’ views and setting common goals:

“*The new generations are very innovative, and they often come up with brilliant ideas…. I always try to examine things through their lens and work with them closely to explore the new practices they suggest.*” (Code M13) [Generation Xer; Nurse Manager]

“*In my own team, I work closely with my colleagues to set common goals, and we brainstorm together on whether any new practices are feasible and desirable.*” (Code M15) [Millennial; Nurse Manager]

### 4.4. Embracing Change and Quality

Most first-line nurse managers recognize that healthcare has undergone tremendous changes and that people have higher expectations of quality of care than before. They emphasized the need to be able to embrace change and ensure quality at work:

“*A nurse manager must be ‘trendy.’ You must be able to catch up with the latest developments in healthcare, its rapid advancements and changes.*” (Code M05) [Millennial; Nurse Manager]

“*The world is changing; nowadays, care recipients regard receiving quality care as their right…. The most important* thing is to cultivate a culture in the work unit that facilitates the pursuit of quality.” (Code M29) [Baby Boomer; Nurse Manager]

#### 4.4.1. Being Adaptable and Flexible to Changes

Some informants shared their views about the need to deal with rapid changes at work owing to rapid advancements in modern technology. This is making it necessary for first-line nurse managers to be knowledgeable about information technology and to be highly adaptable and flexible enough to change:

“*It is vital to keep yourself abreast of the latest development in technology, as in our daily lives, many changes are taking place because of new technological applications. There is also a plan to transform the hospital into a smart hospital.*” (Code M03) [Generation Xer; Nurse Manager]

“*There are many changes at work. For example, the hospital has introduced a new electronic medication dispensing system. There are a lot of hiccups with that, but our nurse manager is not good at managing technological stuff.*” (Code N13) [Millennial; Advanced Practice Nurse]

Generation Xers and Millennials were noted to be more receptive to change, while Baby Boomers tended to be more reluctant to abandon previous work habits and accept new practices:

“*Promoting change is the right thing to do, but for me, it takes time to accept and adapt to change. Some perceptions and practices are ingrained…. I sometimes doubt whether changes are really necessary.*” (Code N07) [Baby Boomer; Advanced Practice Nurse]

“*Some nurses and nurse managers are very resistant to change, and forget that confronting these challenges can indeed bring opportunities for broadening one’s horizons.*” (Code M13) [Generation Xer; Nurse Manager]

#### 4.4.2. Being Committed to Quality

Most first-line nurse managers attached great significance to their role in safeguarding the quality of care. Among them, Baby Boomers tended to uphold the importance of clinical expertise, and they monitored the quality of care through directly engaging in patient care and staff supervision:

“*A nurse manager is like a goalkeeper, and the ultimate goal is quality care. We must have clinical expertise in order to safeguard it, as nursing work deals with life and death.*” (Code M24) [Baby Boomer; Nurse Manager]

“*If you don’t have the clinical knowledge and skills, how can you coach new staff and supervise the nurses in their daily work?*” (Code M01) [Baby Boomer; Nurse Manager]

The views of the Baby Boomers differed somewhat from those of the Generation Xers and Millennials, who emphasized that first-line nurse managers and frontline nurses should carry out their respective roles in administration and providing direct patient care:

“*I know some nurse managers are providing direct patient care and monitoring staff performance closely, and I think this is inappropriate. Frontline nurses and nurse managers have their own roles to play.*” (Code M07) [Generation Xer; Nurse Manager]

“*The younger generation doesn’t like you to be there to monitor and give commands. What you should do is to stand behind them, but not to closely monitor them.*” (Code M10) [Generation Xer; Nurse Manager]

Most nurse informants agreed with the views of the Generation Xer and Millennial nurse managers, saying that they looked for more autonomy at work:

“*Back to the old days when I was very junior, I learned the most from my colleagues but not the nurse manager…. I do think that she (and other nurse managers) should delegate work like staff training and supervision to colleagues.*” (Code N20) [Millennial; Advanced Practice Nurse]

“*The nurse manager requires us to inform him of everything, no matter how big or small. I think he needs to learn to empower colleagues; we can handle most of the work independently.*” (Code N19) [Generation Xer; Advanced Practice Nurse]

## 5. Discussion

Past relevant studies tended to identify an exhaustive list of dimensions or standards of competency and to conclude that nurse managers need to develop multifaceted abilities to attain managerial success [23]. This study focused on capturing the core elements of the competencies of nurse managers, namely staff advocacy, resource allocation, team effectiveness, and change and quality management. This study also uncovered differences in the views of different generations of nurses on what contributes to managerial success in nursing. The findings shed light on how to lead a multi-generational nursing workforce so as to bring about positive staff and patient outcomes.

### 5.1. Discussion of the Findings

Regarding the role of first-line nurse managers in advocating for the interests of staff, previous studies cited a sense of responsibility and fairness as important inner competencies of nurse managers [14,23]. Among the three generations of nurse managers, Baby Boomers are the most committed to their organizations and coworkers and demonstrate the strongest sense of professionalism. Unfavorable work conditions, such as a heavy workload, do not easily dampen their commitment because they view nursing as a calling [17,20]. They are usually “workaholics” and are willing to sacrifice their personal life and interests for the sake of work [19]. In this, they differ from Generation Xers and Millennials, who view nursing as an occupation rather than a profession and constantly strive to achieve a balance between their work life and personal and family interests [17,19]. These findings warrant special attention because those currently in the position may experience distress due to the conflict between their role expectations and their desire to seek a work-life balance [20]. Past studies noted that Baby Boomers tend to get more frustrated by a lack of development and promotional opportunities than those of other generations [17]. This study discovered that Generation Xers and Millennials are more reluctant to seek promotion because they perceive that they will risk losing their “work-life balance” if they take on a senior position [24]. This finding is contradictory to that of previous research, where it was noted that these generations of nurses have high aspirations for leadership positions [25].

Consistent with the findings of other researchers, in this study, resource allocation was identified as a required area of competency for first-line nurse managers and encompasses the elements of the workforce, financial, material, and facility management [23,26]. Previous researchers recognized that most nurse managers had difficulties with work scheduling and role assignments, especially in times of manpower shortage [23]. This study further noted that Generation Xers and Millennials are less confident than Baby Boomers in managing staff assignment issues. Concerning financial management, most nurse managers gave a moderate rating to their ability to manage budgets [16,23], and some perceived themselves as being the least competent at fiscal planning [27,28]. This study further indicated that Generation Xers and Millennials are generally less capable than Baby Boomers in handling fiscal-related matters, such as planning their work unit’s annual expenses.

Communication, collaboration, and interpersonal skills were constantly quoted in previous research as skill sets crucial to the success of nurse managers [12,23]. This study further added insights into the differences in the preferred mode of communication of different generations of nurses. Generation Xers and Millennials were born in an era of rapid technological advancements; when compared with Baby Boomers, they are less interested in direct physical contact with others. They are easily frustrated by lengthy discussions in meetings and prefer immediate feedback and other means of communication (e.g., emails and instant messaging by phone) [17]. In collaborating with others, Millennials, in particular, hate hierarchy. They highly value team cohesion and prefer a collective and collaborative approach at work [19,20]. They believe that everyone can speak for themselves and share their opinions and that all viewpoints should be acknowledged. On the contrary, Baby Boomers and Generation Xers place more stress on respecting and listening to the views of seniors and do not easily embrace the new perspectives of the younger generations [17]. The AONE recognized the influence of generational diversity and emphasized that by capitalizing on differences, a highly effective team can be created [14].

In previous studies, nurse managers cited many instances of engaging in direct patient care. They ranked daily unit operations and supervisory responsibilities highest to ensure the best quality of nursing care [23,26]. This study found that Baby Boomers are fond of the traditional approach of one-on-one coaching. Generation Xers and Millennials, for their part, seek to work independently to demonstrate their competence and prefer learning from peers rather than from nurse managers [17,19]. The findings of this study further showed that Generation Xers and Millennials do not regard clinical expertise as an important area of competency for first-line nurse managers. They want the nurse managers to empowering them to make decisions in daily practice [29]. This is inconsistent with extant studies, which highlight clinical practice knowledge and skills as crucial elements of nurse manager competencies [14,23,28]. Previous researchers criticized the traditional approach of selecting nurse managers based on their level of clinical competence and seniority [30]. They stressed that nurses perceive that they receive better support and are more motivated if nurse managers are more sensitive to their needs, rather than if the nurse managers directly engage in supervising and directing patient care and control the work that is performed [30].

Frontline nurses, in general, agreed that first-line nurse managers play an essential role in monitoring and facilitating changes at work. It was noted that having grown up in a digital era with rapid technological advancements and an explosion of information, Generation Xers and Millennials are good at absorbing new information, multi-tasking, and adapting to change [29]. In comparison, Baby Boomers are usually more rigid, less flexible in adapting to change, and easily distressed by the rapid changes in work processes [16]. Scholars commented that changes in organizational policies and practices should not have diminished the influence and authority of nurse managers and that frontline nurses should be able to derive support not only from the nurse managers but also from the wider organization [30].

### 5.2. Implications of Management Practice and Education

The findings of this study have important implications for future management practice and education. This study found that the first-line nurse manager competencies preferred by the newer generations are different from those of older generations of frontline nurses. Along with the increasing number of Generation Z nurses joining the workforce, the workplace will become more heterogeneous than before in terms of generational diversity. It is believed that nurse managers will face more challenges when working with multiple generations of nurses and in creating a workplace that can ensure team effectiveness while also retaining existing nurses and attracting new entrants [30]. Scholars criticized nurse managers for “continu[ing] to manage the work environment as members of each generation operate from a universal perspective” [30] (p. 720). It should be noted that a “one-size-fits-all” set of management competencies will not address the needs and expectations of a generation-diverse workforce. Both hospital administrators and nurse managers need to be cognizant of the distinct features and values of the different generations of nurses. Future management training should be tailored to increase their knowledge of generational diversity and its influence on staff and patient outcomes [19,20].

Some areas merit immediate attention, namely the misalignment between multi-generation nurse managers and frontline nurses with regard to their preferred means of communication and leadership styles, as well as the reluctance of the younger generations to take on managerial positions. Healthcare organizations need to provide appropriate structures and support, for instance, by creating electronic communication platforms (such as mobile apps) to facilitate team communication while also providing continuing training to both novice and experienced nurse managers [31]. Regarding the preparation of nurse managers, it should be noted that to “throw” them into the position without adequately preparing them is a haphazard approach [28]. Previous researchers noticed that the perceived competence scores of more experienced nurse managers were lower than those of novice nurse managers [28]. Similarly, this study found that Baby Boomer, Generation X, and Millennial nurse managers vary in their work abilities and in the difficulties that they face at work. This indicates that the focus of management training should not be only on developing orientation, mentorship, and succession planning programs for novice nurse managers. Rather, it is necessary to provide structured empowerment programs for nurse managers at different stages in their careers to promote their continued advancement, and the programs must be tailored to meet the generational needs of all parties [28].

### 5.3. Limitations and Further Research

This study was conducted in Hong Kong, an Asian city with a Western healthcare system, and it fell short in examining the experiences of Generation Z nurses. As culture plays a role in shaping generation-specific perspectives, further research on other cultural contexts, as well as on the perspectives of Generation Z nurses, is needed to attain a comprehensive understanding of what contributes to the managerial success of first-line nurse managers. Research examining both the perceptions of nurse managers and frontline nurses while taking generational diversity into account is limited. It should also be noted that modern healthcare is constantly changing and that the competencies critical for managerial success are likely to vary along with such changes. Continuing scholarly efforts are needed to study the dynamics among the multiple generations of frontline nurses and nurse managers, to understand better how generational diversity influences managerial effectiveness and what kinds of generation-specific strategies can bring about positive staff and patient outcomes [16]. Further studies that employ quantitative approaches and involve larger samples are needed to validate the findings of this study.

## 6. Conclusions

The findings of this study indicate that each generation of nurses has its distinct features and values and that each generation’s views of what constitutes the required competencies of first-line nurse managers are contradictory to those of other generations. These differences should prompt hospital administrators and nurse educators to reassess the best strategies for supporting existing and future nurse leaders. First-line nurse managers also need to reflect on their current practices in leading a multi-generational nursing workforce.

## Figures and Tables

**Table 1:** Demographic Characteristics of the Informants.

Total (N = 48)	First-Line Nurse Managers (n = 29)	Frontline Nurses (Subordinates)(n = 19)
	n (%)	
Gender		
Male	7 (24.1)	6 (31.6)
Female	22 (75.9)	13 (68.4)
Age		
30 or below	1 (3.5)	2 (10.5)
31–40	2 (6.9)	8 (42.1)
41–50	8 (27.6)	7 (36.9)
51–60	13 (44.8)	2 (10.5)
61 or above	5 (17.2)	0 (0)
mean ± SD	48.2 ± 7.17	36.2 ± 9.26
Type of generation		
Baby Boomers	6 (20.7)	2 (10.5)
Generation X	18 (62.1)	7 (36.9)
Millennials	5 (17.2)	10 (52.6)
Type of hospital		
Public	25 (86.2)	14 (73.7)
Private	4 (13.8)	5 (26.3)
Type of clinical setting		
Medical	13 (44.8)	10 (52.6)
Surgical	7 (24.1)	7 (36.9)
Others (e.g., out-patient clinics, psychiatry, accident, and emergency)	9 (31.1)	2 (10.5)
Rank		
Ward Manager	29 (100)	-
Advanced Practice Nurse	-	12 (63.2)
Registered Nurse	-	4 (21.0)
Enrolled Nurse	-	3 (15.8)
Years of work experience, mean ± SD	28.20 ± 5.45	18.21 ± 8.13
Years of working as a nurse manager, mean ± SD	8.54 ± 4.27	-

**Table 2:** Interview guide.

**For First-Line Nurse Managers**
1. How do you perceive your role as a first-line nurse manager? 2. Which aspects of work do you find satisfying, and which aspects of work do you find difficult or challenging? 3. From your perspective, what are the attributes of an effective first-line nurse manager? 4. From your perspective, what competencies are crucial for the success of management in healthcare/nursing? 5. What suggestions would you make to develop first-line nurse managers in the future?
**For Frontline Nurses**
1. How do you perceive your experience of working with your first-line nurse manager? 2. How do management practices affect you and your work? 3. How do you perceive the quality of nursing management at the unit level? 4. What kinds of nurse manager competencies do you consider crucial for effective management in healthcare/nursing? 5. What suggestions would you make to improve the quality of nursing management and the training for future nurse leaders?

## Data Availability

The data that support the findings of this study are available from the corresponding author upon reasonable request.
